# Single cell time-lapse analysis reveals that podoplanin enhances cell survival and colony formation capacity of squamous cell carcinoma cells

**DOI:** 10.1038/srep39971

**Published:** 2017-01-06

**Authors:** Tomoyuki Miyashita, Youichi Higuchi, Motohiro Kojima, Atsushi Ochiai, Genichiro Ishii

**Affiliations:** 1 Laboratory of Cancer Biology, Department of Integrated Biosciences, Graduate School of Frontier Sciences, The University of Tokyo, Kashiwa, Chiba, Japan; 2 Division of Pathology, Exploratory Oncology Research and Clinical Trial Center, National Cancer Center, Kashiwa, Chiba, Japan

## Abstract

Tumor initiating cells (TICs) are characterized by high clonal expansion capacity. We previously reported that podoplanin is a TIC-specific marker for the human squamous cell carcinoma cell line A431. The aim of this study is to explore the molecular mechanism underlying the high clonal expansion potential of podoplanin-positive A431cells using Fucci imaging. Single podoplanin-positive cells created large colonies at a significantly higher frequency than single podoplanin-negative cells, whereas no difference was observed between the two types of cells with respect to cell cycle status. Conversely, the cell death ratio of progenies derived from podoplanin-positive single cell was significantly lower than that of cells derived from podoplanin-negative cells. Single A431 cells, whose podoplanin expression was suppressed by RNA interference, exhibited increased cell death ratios and decreased frequency of large colony forming. Moreover, the frequency of large colony forming decreased significantly when podoplanin-positive single cells was treated with a ROCK (Rho-associated coiled-coil kinase) inhibitor, whereas no difference was observed in single podoplanin-negative cells. Our current study cleared that high clonal expansion capacity of podoplanin-positive TICs populations was the result of reduced cell death by podoplanin-mediated signaling. Therefore, podoplanin activity may be a therapeutic target in the treatment of squamous cell carcinomas.

Cancer cells are comprised of phenotypically and functionally heterogeneous cell populations. Cancer stem cells (CSCs), also known as tumor initiating cells (TICs), are the cell subpopulation which are characterized by higher tumorigenic capacity^1^. For these reasons, TICs are considered to be the underlying cause of tumor recurrence, metastasis and development of drug resistance^2^,^3^. TICs have been identified in many human tumors including leukemia^4^, breast^5^, brain^6^, prostate^7^,^8^, colon^9^, and pancreas cancers^10^. The most common experimental methods for TICs identification are *in vivo* xenotransplantation into immunocompromised mice and/or *in vitro* sphere formation and colony formation assays^11^.

Cell surface markers are widely used for isolation of normal or cancer stem cells. Until now, many TICs markers including CD44^12^,^13^, CD133^14^,^15^, Lgr5^16^ and more were identified. We previously reported that cell surface marker Podoplanin (PDPN), a mucin-like transmembrane glycoprotein, is a TIC marker of the human squamous cell carcinoma cell line, A431^17^. In cancer cells, PDPN enhances the tumor metastatic potential by eliciting tumor cell-induced platelet aggregation through activation of the platelet receptor, CLEC-2 (C-type lectin-like receptor 2)^18^. Furthermore, the ability of PDPN to interact with member of the ERM (ezrin, radixin, moesin) protein family^19^ promotes tumor cell motility^20^, invasion^21^, and metastasis^22^. PDPN-positive (PDPN^+^) A431 cells had higher tumorigenicity and clonogenicity than PDPN-negative (PDPN^−^) A431 cells^17^. Rhadinani *et al*. reported that in esophageal squamous cell carcinoma cell lines, PDPN^+^ cells displayed high tumorigenicity and clonogenicity, concluding that PDPN is an efficient TIC marker^23^.

*In vitro* single cell clonogenic assays are commonly deployed for examining the cytotoxic effects of radiation and/or drug treatment^24^,^25^. This technique can also be used for the *in vitro* evaluation of the survival and proliferative capabilities of cancer cells. This approach can also be used to characterize TICs, as the size of colonies, i.e., the number of grown cells, derived from single cells are indicators of the clonogenicity of the seeded cells. A crucial challenge is to examine how single TIC and non-TIC cells grow in a time-dependent manner and why single TICs can create large colonies at a higher frequency compared to single non-TICs.

To overcome this problem, we used single cell based live-imaging based on the Fucci (fluorescent ubiquitination-based cell cycle indicator) system to visualize the differences between PDPN^+^ and PDPN^−^ cancer cells, with respect to cell cycle status, viability, and death.

## Results

### Cell fate map of single A431/Fucci2

**Figure 1:**
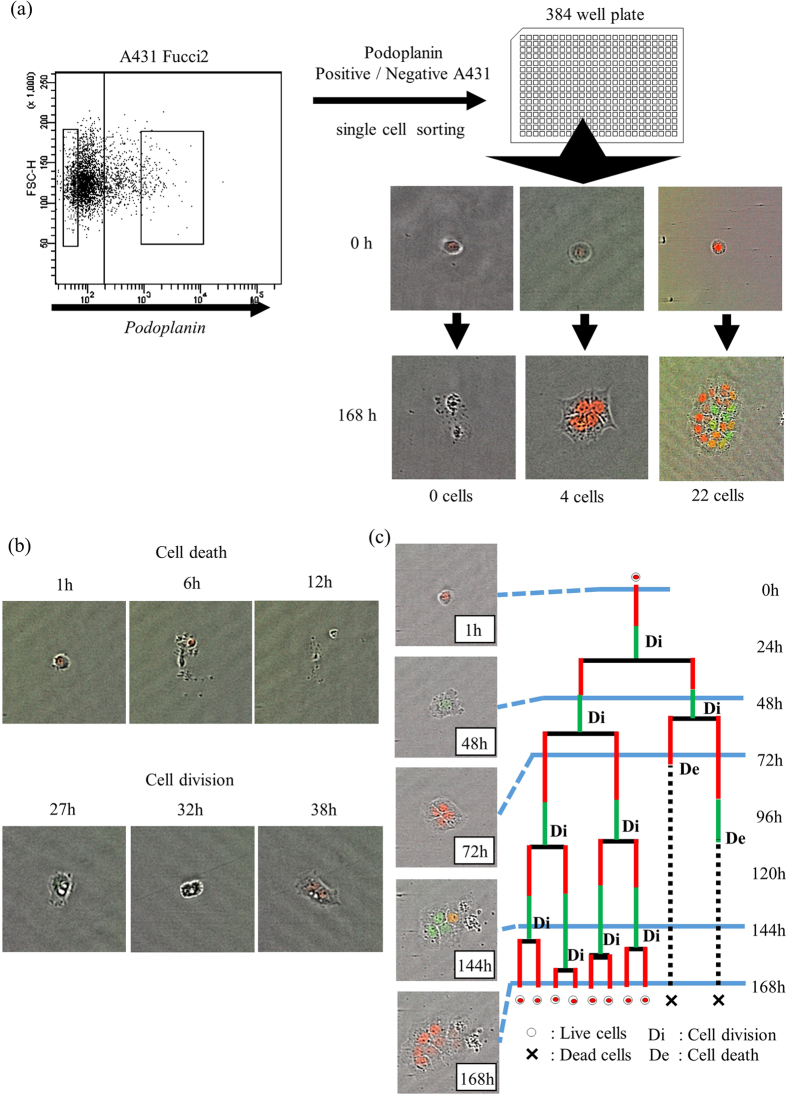
Schema of the experiment. (**a**) PDPN expression of A431/Fucci2 cells (upper left dot plot). Single PDPN^+^ or PDPN^−^ A431/Fucci2 cells was sorted and cultured in 384 well plate. Left panel; No viable cells were observed after 7 days. Middle panel; Single sorted cell created a “small” colony consisting of four progenies. Right panel; Single sorted cell created a “large” colony consisting of twenty-two progenies. (**b**) Typical morphological appearances of “cell death” and “cell division”. Upper panel; At 6 h to 12 h culture time, reduction of cellular volume, and cell bursts were observed, which defined as “cell death”. Lower panel; At 32 h culture time, cleavage furrow formatted and separated two cells both of which have red nuclear fluorescence were observed at 38 h (cell division). (**c**) Cell fate map of A431/Fucci2 cells. Based on Fucci fluorescent, cell cycle status, cell division and cell death were identified. Length of the red lines and the green lines reflected the length of G0/G1 phase and the length of S/G2/M phase, respectively.

We seeded single PDPN^+^ and PDPN^−^ A431/Fucci2 cells into a 384-well plate. After 7 days in culture, various number of cells were found in each well (Fig. 1a). Time-lapse imaging of the culture throughout the 7-day incubation period allowed us to calculate the “cell death” and “cell division” ratios (Fig. 1b, upper and lower panel, respectively). Moreover, the cell cycle state of each cell was determined by the color of its nuclear fluorescence. Using these methods, we created a “cell fate map” where the cell cycle phase, cell division and cell death of all grown cells are displayed (Fig. 1c). In the example presented in Fig. 1c, the initial cell divided and produced two daughter cells. One daughter cell continued growing and finally produced eight live cells, whereas the other cell divided once and the two granddaughter cells died. The red and green lines represent the length of the G0/G1 and S/G2/M phases, respectively.

### Growth rates of single PDPN^+^- and PDPN^−^- derived cells

**Figure 2:**
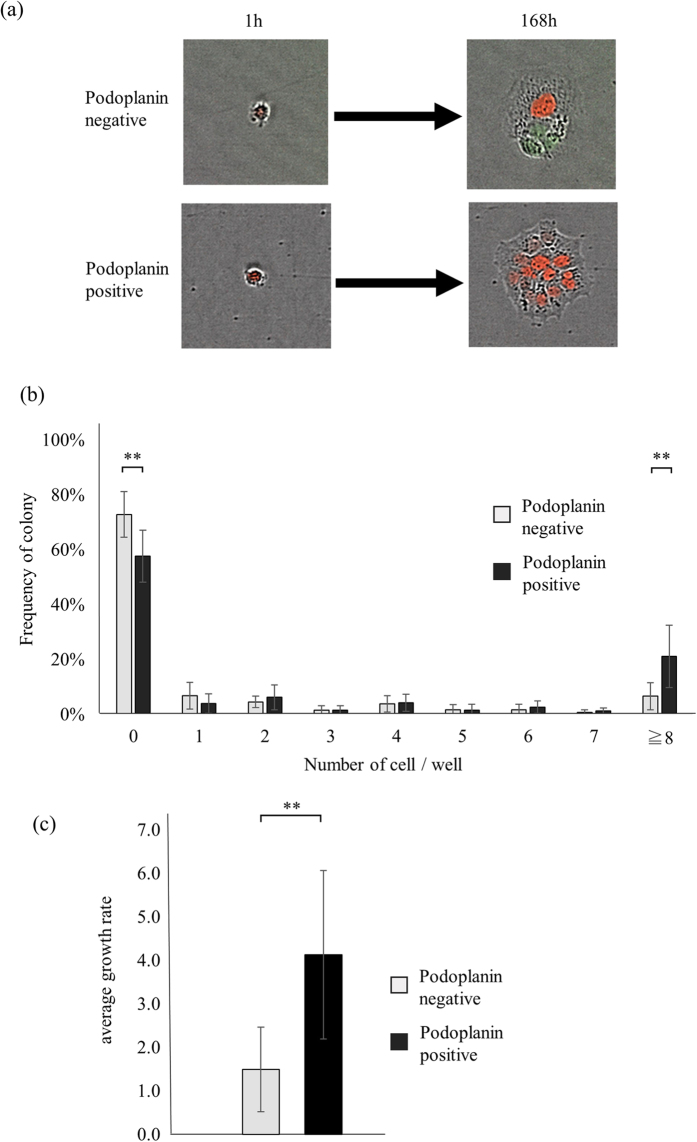
Growth of single PDPN^+^- and PDPN^−^- derived cells. (**a**) Representative samples of colonies at the 7th day. Upper panel; a single PDPN^−^ A431 cell created a small colony consisting of three cells. Lower panel; a single PDPN^+^ A431 cell created a large colony consisting of 14 cells. (**b**) The number of live progenies per well after 7 days in culture. Results of five experiments are summarized. (**c**) Growth rates. Values are means ± S.D. from five independent experiments. Statistical analysis was performed using Student’s t-test. **p < 0.05.

Representative samples of colonies generated within 7 days starting with single sorted PDPN^−^ and PDPN^+^ A431 cells are displayed in the upper and lower section of Fig. 2a, respectively. The results of 5 experiments are summarized in Fig. 2b. The percentages of wells seeded with single PDPN^−^ or PDPN^+^ cells that lacked live cells at the end of the 7-day incubation period were 73 ± 4% and 58 ± 4%, respectively. Only 7 ± 2% of the single initial PDPN^−^ cells created large colonies (≥8 cells) vs. 21 ± 5% of PDPN^+^ cells, with the difference being statistically significant (*p* = 0.03). We calculated growth rate of single PDPN^+^ and PDPN^−^ cells (See Material and Methods). Overall, seeded PDPN^+^ single cells exhibited a significantly higher growth rate compared to PDPN^−^ cells (4.1 ± 0.9 vs. 1.5 ± 0.4, *p* = 0.03) (Fig. 2c). Furthermore, we used other squamous cell carcinoma cell line, NCIH2170 cell (human lung squamous cell carcinoma) and obtained a similar result (Sup. Fig. 2). The average growth rate of PDPN^+^ and PDPN^−^ NCIH2170 cells were 8.0 ± 0.9 and 5.2 ± 0.3, respectively (*p* = 0.006). The frequency of large colonies (≧8 cells/well) were 37 ± 2% in PDPN^+^ and 30 ± 1% in PDPN^−^, respectively (*p* = 0.008). Taken together, PDPN^+^ cell had higher growth rate and large colony forming ability than PDPN^−^ cell.

### Cell cycle analysis of PDPN^+^- and PDPN^−^- derived cells

**Figure 3:**
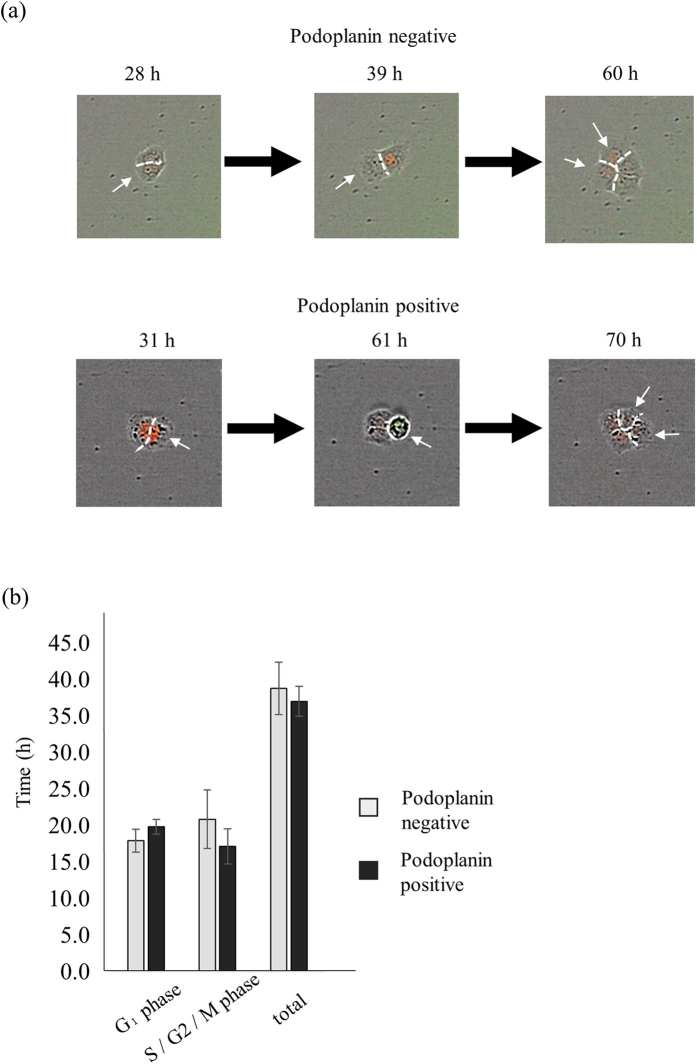
Cell cycle analysis of PDPN^+^- and PDPN^−^- derived cells. (**a**) Representative images of cell cycle progression of single A431 PDPN^−^ (upper panel) and PDPN^+^ (lower panel) cells. Upper panel; two mCherry-positive cells, derived from a single PDPN^−^ cell, were detected at 28 h (left). Cells indicated with a white arrow had their nuclear fluorescence changed from red to green at 39 h (middle). At 60 h, one green cell (indicated by a white arrow) produced two red cells (right). Lower panel; two mCherry-positive cells, derived from a single PDPN^+^ cell, were detected at 31 h (left). Cells indicated with a white arrow had their nuclear fluorescence changed from red to green at the time of 61 h (middle). At 70 h, one green cell (indicated by a white arrow) produced two red cells (right). (**b**) Cell cycle status of progenies of PDPN^+^ and PDPN^−^ cells. Values are means ± S.D. from five independent experiments. Statistical analysis was performed using Student’s t-test.

We compared the cell cycle status between cells derived from PDPN^+^ and PDPN^−^ seeded cells by measuring the duration of the periods when each nucleus displayed red (G0/G1 phase) and green (S/G2/M phase) fluorescence. Representative examples of cell cycle progression of A431/Fucci2 cells derived from single PDPN^−^ and PDPN^+^ cells are displayed in the upper and lower portions of Fig. 3a, respectively. In the case of wells seeded with PDPN^−^ cells, two mCherry-positive cells were produced after 28 h. In one cell (indicated by a white arrow in the left and middle panels of Fig. 3a), nuclear fluorescence had changed from red to green at 39 h. At 60 h, the green cell had separated into two red cells (indicated by a white arrow in the right panel of Fig. 3a).

With respect to the cell cycle progress of PDPN^+^ cells, at 31 h two mCherry-positive cells had been produced. At 61 h, in one cell (indicated by a white arrow), nuclear fluorescence had changed from red to green. At 70 h, the green cell had separated into two red cells (indicated by a white arrow). In total, we analyzed 501 PDPN^−^and 1092 PDPN^+^ cells. The average G1 phase durations of PDPN^+^- and PDPN^−^- derived cells were 19.9 ± 0.5 h and 18.0 ± 0.7 h, respectively, whereas the respective average durations of S/G2/M phases were 17.2 ± 1.1 h and 20.9 ± 1.8 h. There was no statistically significant difference in average doubling time between A431 PDPN^+^- and PDPN^−^- derived cells (37.0 ± 0.9 h vs. 38.8 ± 1.6 h) (Fig. 3b).

### Cell death ratio of PDPN^+^- and PDPN^−^- derived cells

**Figure 4:**
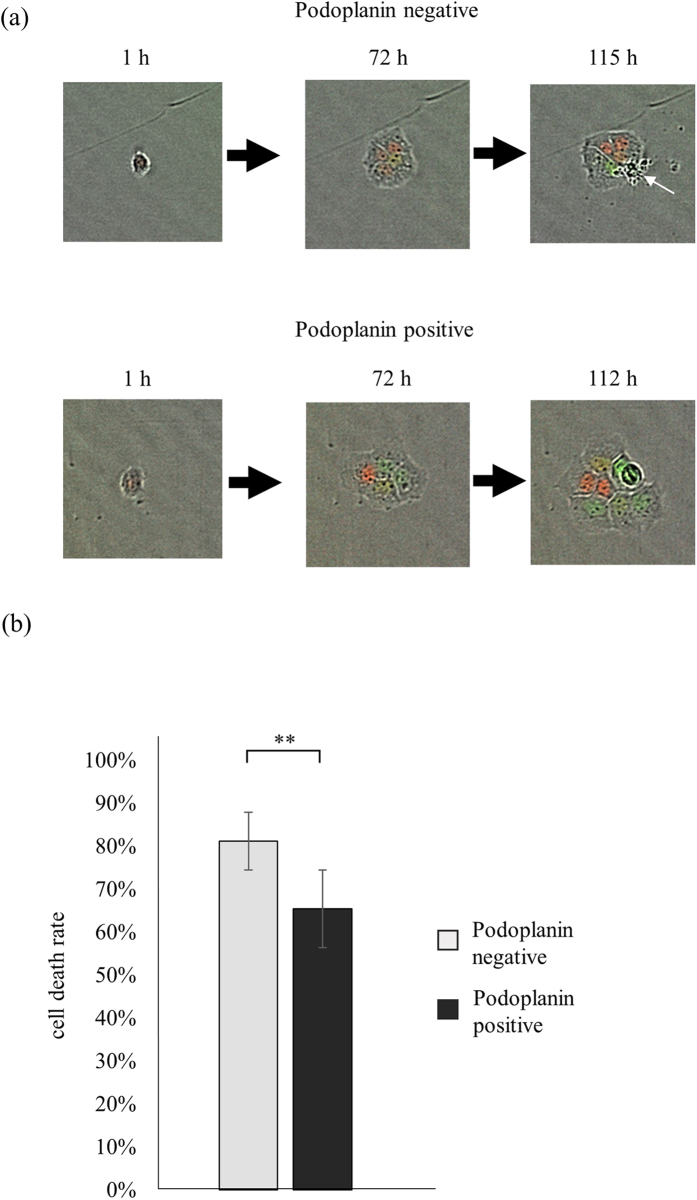
Cell death of PDPN^+^- and PDPN^−^- derived progenies. (**a**) Representative cell death images of cells derived from a single PDPN^−^ A431 cell (upper panel) and a single PDPN^+^ A431 cell (lower panel). Upper panel; four PDPN^−^- derived cells were alive at 72 h (middle), but one cell (indicated with a white arrow) died at 115 h (left panel). Lower panel; PDPN^+^- derived cells did not die during cell divisions. (**b**) Calculated cell death ratios. Values are means ± S.D. from five independent experiments. Statistical analysis was performed using Student’s t-test. **p < 0.05.

Based on the cell fate map (Fig. 1c), we calculated cell death ratio of each well (See Material and Methods). The upper panel of Fig. 4a indicated that PDPN^−^ single A431 cell often caused cell death during the culture (with white arrow). On the other hand, PDPN^+^ single A431 cell did not cause any cell death during cell divisions. The results are summarized in Fig. 4b. 65 ± 4% of PDPN^+^- derived cells caused cell death, however; 81 ± 3% of PDPN^−^- derived cells caused cell death (*p* = 0.01). Consequently, PDPN^+^ cells caused cell death less often than PDPN^−^ cells, which resulted in higher growth ratio.

### Cell cycle status and cell death rates of PDPN^+^- and PDPN^−^- derived cells of each generation

**Figure 5:**
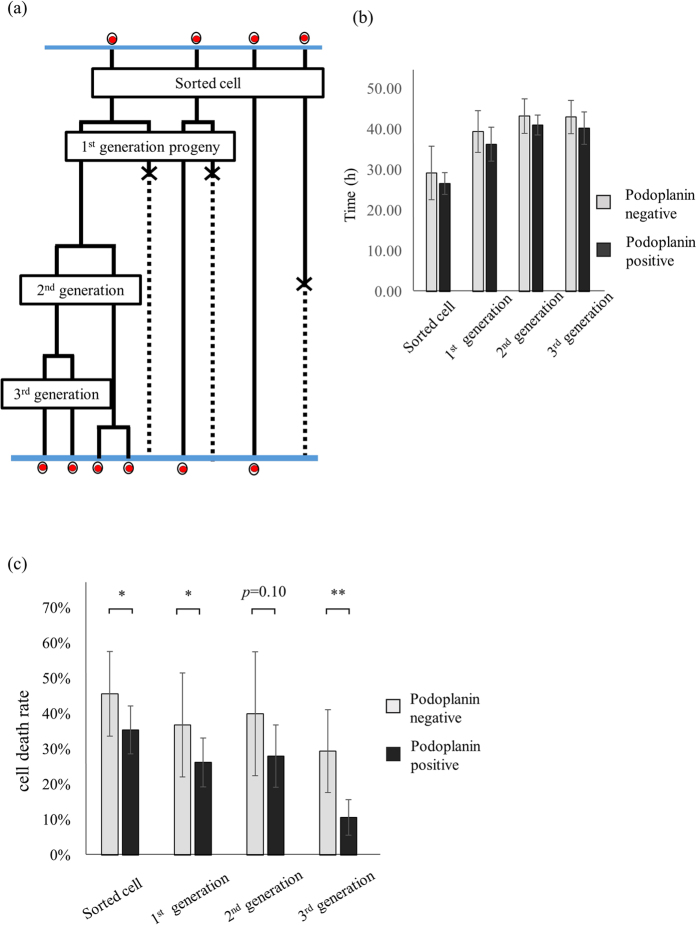
Cell cycle status and cell death ratio of each generation. (**a**) Cell fate map focusing on each generation. In the beginning, there were four “sorted cells”. Two of them divided and produced four daughter cells, defined as “1st generation progenies”. Two the four cells died, while one divided into two new cells, defined as “2nd generation progenies”. This cell divided, giving the “3rd generation progeny”. (**b**) Cell cycle status of progeny of each generation, deriving from single PDPN^+^ and PDPN^−^ cells. (**c**) Cell death ratios of grown cells of each generation. Values are means ± S.D. from five independent experiments. Statistical analysis was performed using Student’s t-test. *p < 0.1, **p < 0.05.

We determined the cell cycle status and death ratio of each generation of cells derived from the single seeded cells (Fig. 5a). There was no significant difference in the average doubling time between A431 PDPN^+^ and PDPN^−^ progeny cells of each generation (Fig. 5b). We calculated the cell death ratio of grown cells of each generation. PDPN^−^-derived cells were more prone to dying compared to PDPN^+^- derived cells until 2^nd^ generation progenies. With respect to 3^rd^ generation progenies, the cell death ratio of PDPN^−^- derived cells was significantly higher than that of PDPN^+^-derived cells (11 ± 2% vs. 29 ± 5%, *p* = 0.01) (Fig. 5c).

### Knockdown of PDPN in A431 cells suppresses colony-forming ability

In order to investigate whether or not PDPN has a functional role in colony forming ability, we transfected shPDPN expression vectors into A431/mCherry-hCdt1 cells. PDPN expression in transfected A431 cells was confirmed by flow cytometric analysis (Sup. Fig. 3). After a 7-day incubation, 61 ± 4% of cells transfected with shLuc had not produced any living cells. Cells transfected with shPDPN1 or shPDPN3 exhibited higher percentages, 88 ± 7% and 78 ± 9%, respectively. The difference between shLuc and shPDPN1 was statistically significant (*p* = 0.006), as was also the difference between shLuc and shPDPN3 (*p* = 0.05).

**Figure 6:**
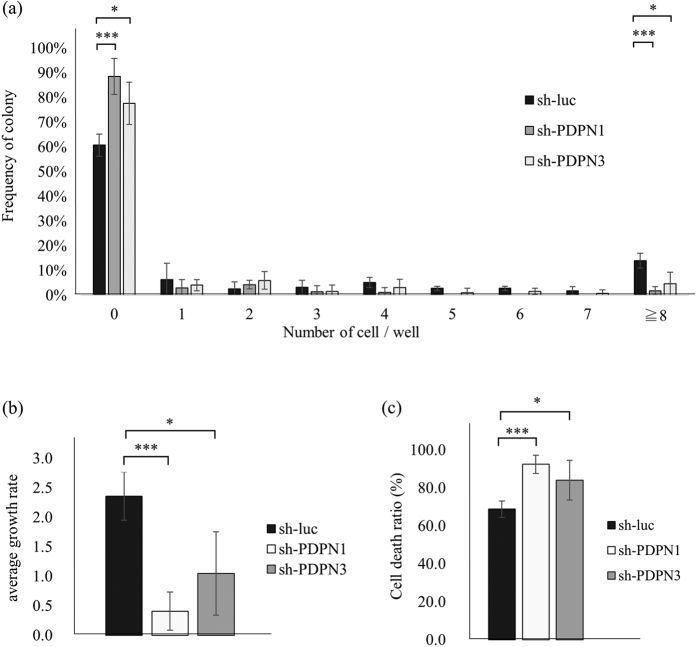
Sh-RNA transfected A431 cells. (**a**) The number of living cells per well of shRNA-transfected A431 cells after 7 days in culture. The results of three experiments are summarized. (**b**) Growth rate of shRNA-transfected A431 cells. (**c**) Cell death ratios of shRNA-transfected A431 cells. T Results of three experiments are summarized. Values are means ± S.D. from three independent experiments. Statistical analysis was performed using Student’s t-test. *p < 0.1, ***p < 0.01.

The percentage of cells that created large colonies was higher in shLuc-transfected cells (14 ± 3%) compared to shPDPN1- (only 2 ± 2%) and shPDPN3- (4 ± 6%) transfected cells, while both differences were statistically significant (shLuc vs. shPDPN1, *p* = 0.006; shLuc vs. shPDPN3, *p* = 0.04). Moreover, as seen in Fig. 6b, control cells displayed a significantly higher growth rate (2.4 ± 0.4) compared to shPDPN1-transfected cells (0.7 ± 0.3, *p* = 0.003) and shPDPN3- transfected cells (1.1 ± 0.7, *p* = 0.05).

With respect to cell death ratios, as seen in Fig. 6c, both shPDPN1- and shPDPN3- transfected cells displayed higher (92 ± 5% and 84 ± 11%, respectively) compared to those of shLuc-transfected cells (69 ± 4%). Both differences were statistically significant (shLuc vs. shPDPN1, *p* = 0.003; shLuc vs. shPDPN3, *p* = 0.08).

### ROCK inhibition decreases the colony forming ability of PDPN^+^ A431 cells

**Figure 7:**
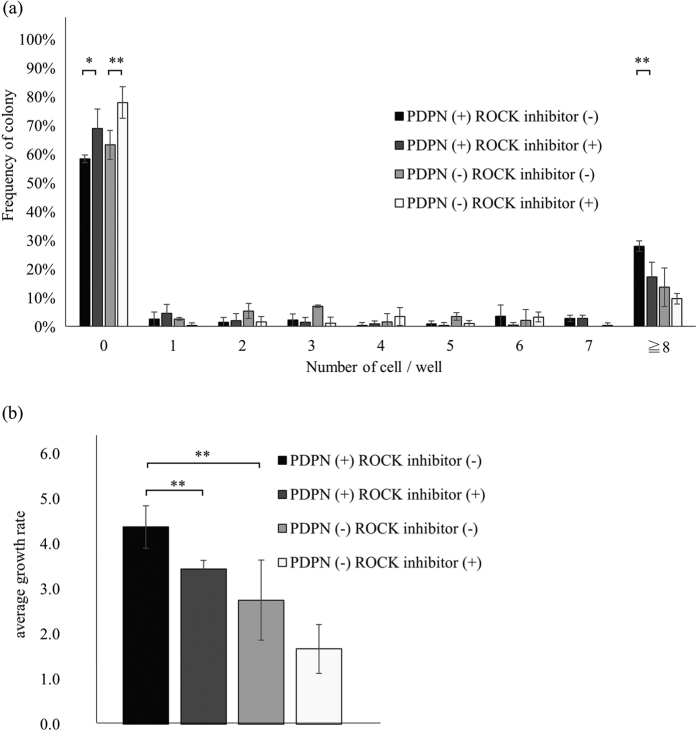
ROCK inhibitor treated A431 cells. (**a**) The number of living cells per well after 7 days in culture, in the presence of a ROCK (Rho-associated coiled-coil kinase) inhibitor. (**b**) Growth rate of ROCK inhibitor treated A431 cells. Results of three experiments are summarized. Values are means ± S.D. from three independent experiments. Statistical analysis was performed using Student’s t-test. **p < 0.05.

PDPN possesses an ERM binding domain and can activate the Rho-ROCK pathway^19^. We investigated whether or not this pathway is necessary to the increased colony forming capacity of PDPN^+^ cells. As seen in Fig. 7a, the use of a ROCK inhibitor increased the percentage of single PDPN^+^ cells that were unable to produce living cells from 58 ± 1% (controls) to 69 ± 7%, with this being more prone to increase (*p* = 0.06). Moreover, ROCK inhibition reduced the percentage of PDPN^+^ cells that were able to give large colonies from 28 ± 2% (controls) to 17 ± 5%, with the decrease being statistically significant (*p* = 0.03). Overall, as seen in Fig. 7b, inhibition of ROCK significantly affected the growth ratio of PDPN^+^ A431 cells (3.4 ± 0.1 vs. 4.4 ± 0.5 in controls, *p* = 0.03). With respect to PDPN^−^ cells, ROCK inhibition did not affect the frequency of large colonies (Fig. 7a; 14 ± 7% vs. 10 ± 2%, *p* = 0.38), or the growth ratio (Fig. 7b; 1.7 ± 0.5 vs. 2.8 ± 0.9 in controls, *p* = 0.15).

## Discussion

Previous studies have used *in vitro* single cell clonogenic assays for evaluating the tumor-initiating capacity of cancer cells. However, the molecular mechanism underlying the high colony-forming efficiency of TICs was not clarified. In order to assess this mechanism, we performed a time-dependent study using single cell time-lapse microscopy to trace the fate of growing cells in cultures derived from single Fucci-transfected cells, throughout a culture incubation period of seven days.

In this study PDPN^+^- derived progeny exhibited lower cell death ratios than PDPN^−^- derived cells. Furthermore, our PDPN knock down experiments strongly suggest that PDPN is a necessary part of the mechanism underlying the increased survival rates of TICs. This may be explained by the fact that PDPN has the ERM binding domain, which allows it to interact with the ERM family proteins. It has been reported that this interaction allows PDPN to regulate the activity of RhoA^19^, which is a small GTPase whose pathway plays a role in cytoskeletal regulation and cell migration. Members of the Rho-associated coiled-coil kinase (ROCK) family are major downstream effectors of RhoA. Many studies have shown that the inhibition of ROCK results in increased apoptosis^26^. In this study, we found that PDPN-knockdown cells were more prone to dying compared to control cells. Moreover, treatment of PDPN^+^ cells with a ROCK inhibitor significantly decreased their colony forming capacity. Although not significant, ROCK inhibitor tended to suppress the growth ratio of PDPN^−^ cells. This may be the result that other signals other than PDPN-mediated may stimulate ROCK pathway even in podoplanin-negative A431 cells. Our results strongly suggest that PDPN-ROCK signaling plays an important role in cell survival and the ability of cells to create large colonies.

TICs are considered as a special cancer cell subpopulation located at the top of the differentiation hierarchy of tumor organization^27^,^28^. In this study, PDPN^+^ A431 cells were found to express higher levels of the undifferentiated-state markers, CD44 and Sox2, but lower levels of the differentiated-state markers, IVL and KRT4 (Sup. Fig. 4). These results indicate that PDPN^+^ cells represent a less differentiated state than PDPN^−^ cells, which is consistent with the immunohistochemical observation that PDPN^+^ cells are localized in the periphery of the squamous cell carcinoma tumor nests^29^,^30^. Thus, PDPN expression probably reflects a less mature status in the differentiation progress of squamous cell carcinomas.

Many studies have reported that TICs in cancer tissues are predominantly in a dormant state. Devon *et al*. reported that breast cancer cells displaying stem-like characteristics expressed two dormancy-related genes, *TGFB2* and *CDKN1B*^31^. Moreover, it has been reported that sphere-derived lung cancer cells overexpress stem cell markers and display a quiescent phenotype^32^. However, Federica *et al*., studying an *in vivo* tumor xenograft model, reported that CD133-positive colon TICs were primarily associated with increased Ki-67 staining^33^, whereas lung cancer TICs with high expression levels of both ALDH and CD44 were reportedly characterized by a high G2/M phase fraction^34^. To sum up, there is no consensus regarding the cell cycle status of TICs. In the current study, no significant difference in cell cycle status was observed between PDPN^+^- and PDPN^−^-derived cells. It is generally accepted that the cell cycle status of cancer cells is dependent on both intrinsic (cancer cell-derived) and extrinsic (stromal cell-derived) factors present within the cancer microenvironment^35^,^36^,^37^. Our study analyzing the grown cells from single cancer cell might purely indicate the endogenous character of TICs.

Our results clearly indicate that PDPN is responsible for the reduced cell death ratio observed in TICs (PDPN^+^), which allows the formation of large colonies by these cells at frequencies higher compared to non-TICs (PDPN^−^). It is well known that PDPN expression is increased in various malignant tumors including squamous cell carcinomas. PDPN on cancer cell surfaces plays a role in cancer cell-induced platelet aggregation. Platelet aggregation-stimulating (PLAG) domains of PDPN are involved in binding to its platelet receptor CLEC-2. Recent studies reported that the use of neutralizing antibodies targeting the PLAG domain of PDPN can inhibit platelet aggregation by blocking PDPN-CLEC2 interaction, resulting in the suppression of tumor growth and metastasis^38^,^39^, whereas a non-neutralizing anti-PDPN antibody also has been successfully used to suppress tumor development and hematogenous lung metastasis via antibody-dependent cellular cytotoxicity (ADCC) and complement-dependent cytotoxicity (CDC)^40^. Our data suggest that the inhibition of PDPN-ROCK signaling can suppress tumor formation itself. Therefore, one may conclude that PDPN plays a dual role in tumor progression and tumor metastasis, via the activation of the ROCK pathway in cancer cells and via platelet aggregation. Moreover, our results suggest that PDPN activity may be a therapeutic target for suppressing squamous cell carcinomas in the early stages of metastasis.

## Methods

### Cell culture

The human squamous cell carcinoma line A431 and lung squamous cell carcinoma line NCIH2170 were obtained from the ATCC (American Type Culture Collection, America) nonprofit organization. A431 cells were cultured in Dulbecco’s modified Eagle’s medium (DMEM) from Sigma (St. Louis, MO, USA) and NCIH2170 were in Roswell Park Memorial Institute (RPMI) 1640 Medium from Sigma (St. Louis, MO, USA), supplemented with 10% fetal bovine serum (Sigma), and 1% penicillin and streptomycin (Sigma). Cells were incubated at 37 °C in an atmosphere containing 5% CO_2_.

### Generation of A431/Fucci2 cells

CSII-EF-MCS vectors encoding mCherry-hCdt1 (30/120) or mVenus-hGeminin (1/110) fusion proteins (kindly provided by Dr. Miyawaki of the RIKEN Brain Science Institute^41^) were co-transfected with the packaging plasmid (pCAG-HIVgp), and the VSV-G- and Rev-expressing plasmid (pCMV-VSV-G-RSV-Rev) (also provided by the RIKEN Institute) into A431 cells. Lentiviral transfection was performed as previously reported^42^.

A431 cells expressing both mCherry-hCdt1 and mVenus-hGeminin fusion proteins, hereafter referred to as A431/Fucci2 cells, were created by first inserting the mCherry-hCdt1 construct. The fluorescence produced by the mCherry-hCdt1 fusion protein was used to sort successfully transfected cells through FACS using an Aria II cell sorter (BD Bioscience, San Jose, CA, USA). The sorted A431/mCherry-hCdt1 cells were then transfected with the mVenus-hGeminin construct. Finally, mVenus expressed cells were sorted and named A431/Fucci2 cells.

### Generation of PDPN knockdown A431 cells

Two different short hairpin RNA expression constructs, shPDPN1 and shPDPN3, were created as previously described^42^, whereas shRNA specific for the luciferase gene (shLuc) was used as a control.

The lentiviruses were produced in 293 T cells. The Lipofectamine 2000 reagent (Invitrogen, Carlsbad, CA, USA) was used for transfection according to the manufacturer’s instructions. Virus-containing medium was filtered through a 0.45-μm filter and 8 μg/ml of polybrene (Santa Cruz) was added for A431/mCherry-hCdt1 cells.

### Cell cycle analysis

1.0 × 10^6^ A431/Fucci2 cells were fixed in cold 70% Et-OH and suspended in 500 μL of PBS/3% FBS, to which 2 μL of DRAQ5 far-red fluorescent DNA dye (Biostatus, Shepshed, UK) was added. Three fractions of cells were sorted using FACS: mCherry positive - mVenus negative, 2) mCherry positive - mVenus positive, and 3) mCherry negative - mVenus Positive A431/Fucci2 cells. Subsequently, sorted cells were again fixed and stained with DRAQ5, and the DNA content of each fraction was analyzed using the FL3 filter of FACS caliber (BD Bioscience). The resulting histograms were merged (Sup. Fig. 1a). Fluorescent images of A431/Fucci2 cells are shown in Sup. Fig. 1b.

### Single cell sorting and culture

1.0 × 10^6^ of cultured A431/Fucci2 cells were suspended in 100 μL of PBS/3% FBS. The suspension was stained with anti-podoplanin mouse IgG (gp36, clone 18H5, Abcam, Cambridge, UK) or control mouse IgG. The primary antibody was detected with goat anti-mouse IgG PE-Cy7 conjugated antibody. Stained cells were washed and resuspended. FACS Aria II was used to sort PDPN^+^ and PDPN^−^ cells. Single cells of either type were seeded into wells of a 384-well plate (BD Bioscience). First, we performed single cell culture without conditioned medium, but almost of seeded cells caused cell death and little colonies could be observed after 7 days culture. We previously found that the efficient growth of single cancer cell required condition medium factors produced by cancer cells themselves^43^. Therefore, we used conditioned medium of A431 in this experimental setting. 1.0 × 10^6^ of A431/Fucci2 cells were seeded into a 10 cm dish. Two days later, the medium was replaced fresh growth medium. After 24 h, the supernatant was collected and filtrated through a 0.45-μm filter. 100 μL of collected supernatant were added to each well, to which sorted single A431 PDPN^+^ or PDPN^−^ cells were subsequently seeded. Plates were incubated in IncuCyte ZOOM (Essen BioScience, Ann Arbor, MI, USA) and time-lapse images of all 384 wells were obtained every one hour for 7 days. The precise data of the corresponding numbers of seeded and grown cells are provided in Sup. Tables 1, 2, and 3. The ROCK (Rho-associated coiled-coil kinase) inhibitor Y-27632 (Wako Pure Chemical Industries, Wako, Japan) was added into the culture medium at a final concentration of 10 μM.

### Time-lapse imaging of single PDPN^+^ and PDPN^−^ cells

Through time-lapse imaging and analysis of the acquired images, we traced all single initial cells and their progeny, which either underwent cell division and proliferated, or died. Moreover, the Fucci fluorescence technology allowed us to determine the length of each cell cycle phase. Cells at the G0/G1 phases displayed red nuclear fluorescence, due to the accumulation of the mCherry-hCdt1 fusion protein in the nucleus, whereas cells at the S/G2/M phages displayed green fluorescence, due to the accumulation of the mVenus-HGeminin protein during these phases. Burst cells, shrunk cells displaying nuclear fragmentation, and inflated cells exhibiting swelling of organelles, plasma membrane rupture and subsequent loss of intracellular contents, were all defined as types of “cell death”^44^ (Fig. 1b, upper). In contrast, cells whose nuclear fluorescence changed from red to green, as well as cells in the process of separation, were defined as cells undergoing “cell division” (Fig. 1b, lower).

### Calculation of growth rate

The following formula was used to calculate the growth rate for each experiment:


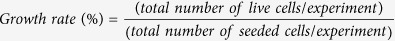


### Calculation of cell death ratio

The following formula was used to calculate the cell death ratio for each well:


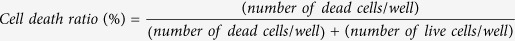


For example, the single sorted cell of Fig. 1c divided into two daughter cells. One of these two cells divided, but both of its progeny died. The other cell gave eight live cells after three divisions. In this case, the number of live cells at the end of the experiment was eight, whereas we had observed the death of two cells. By applying the aforementioned formula, the death ratio is calculated at 20%.

### Real-time reverse transcription polymerase chain reaction (RT-PCR)

Sorted PDPN^+^ and PDPN^−^ cells were suspended in 1 mL of TRIzol reagent (Life Technologies, Carlsbad, CA, USA) and stored at −80 °C. Total RNA was purified from thawed samples through standard techniques, and cDNA was synthesized using the PrimeScript RT reagent Kit (Takara, Shiga, Japan), according to the manufacturer’s instructions. Real time PCR was performed in a Smart Cycler System (Takara) using the SYBR Premix Ex Taq II (Takara), according to the manufacturer’s instructions. The sequences of the used primers are displayed in Sup. Table 4.

## Additional Information

**How to cite this article:** Miyashita, T. *et al*. Single cell time-lapse analysis reveals that podoplanin enhances cell survival and colony formation capacity of squamous cell carcinoma cells. *Sci. Rep.* **7**, 39971; doi: 10.1038/srep39971 (2017).

**Publisher's note:** Springer Nature remains neutral with regard to jurisdictional claims in published maps and institutional affiliations.

## Supplementary Material

Supplementary Figures and Tables
