# Supplementary material for: Single cell time-lapse analysis reveals that podoplanin enhances cell survival and colony formation capacity of squamous cell carcinoma cells
Source: Sci Rep. 2017 Jan 6;7:39971. doi: 10.1038/srep39971 (PMC5216406; doi:10.1038/srep39971)
Supplement: Supplementary Figures and Tables [file srep39971-s1.pdf]

Supplementary material for the manuscript:

Title: Single cell time-lapse analysis reveals that podoplanin enhances cell survival and colony formation capacity of squamous cell carcinoma cells

Authors: Tomoyuki Miyashita, Youichi Higuchi, Motohiro Kojima, Atsushi Ochiai and Genichiro Ishii

(a)

(b)

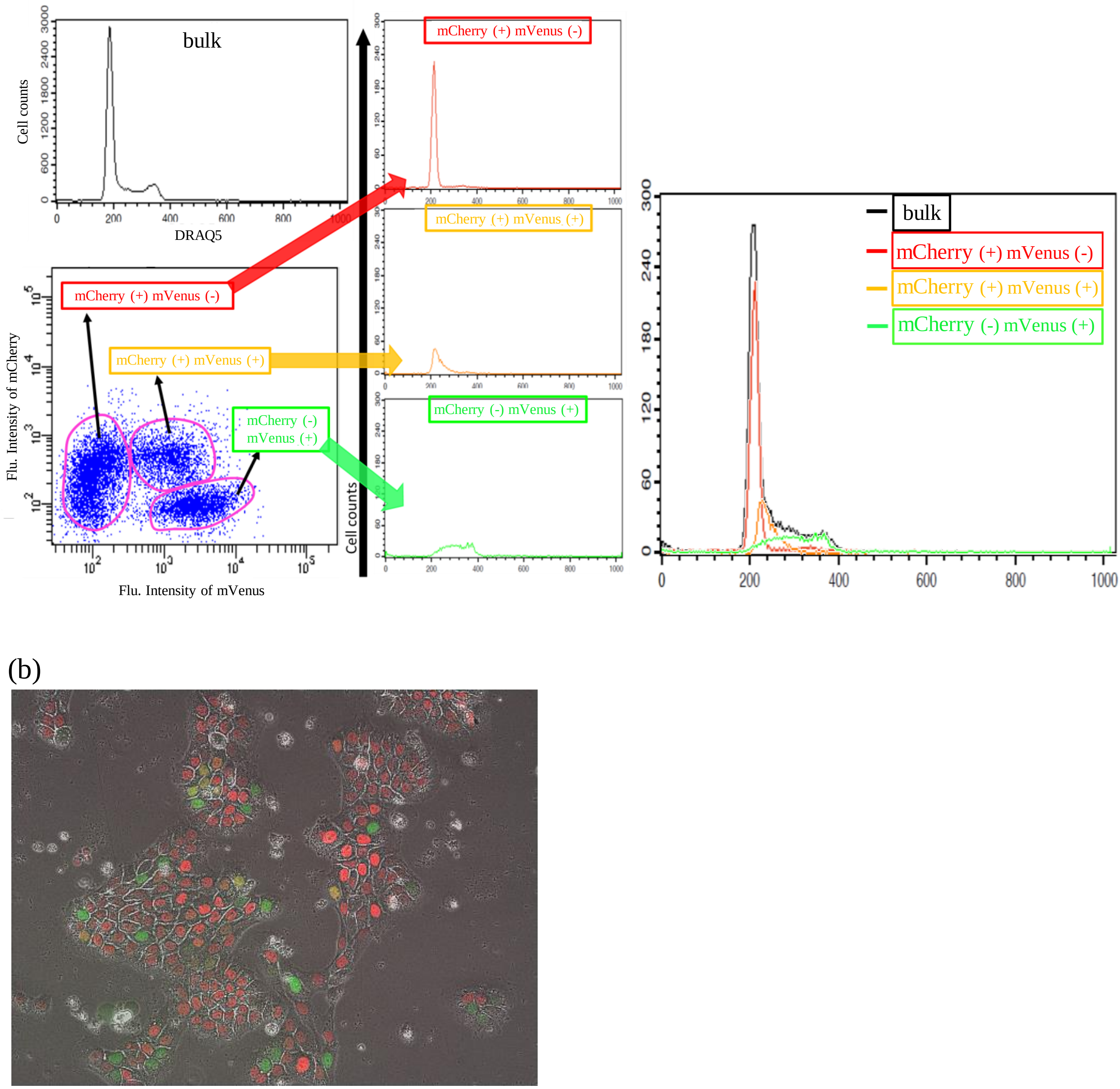

Supplementary Figure 1

Generation of A431/Fucci2 cells

(a) The cell cycle histogram. DNA contents were stained with DRAQ5 and measured. (b) Fluorescence images of A431/Fucci2 cells. Red fluorescent cells were at the G1 phase, yellow fluorescence cells at early S phase, and green fluorescence cells at the S/G2/M phase.

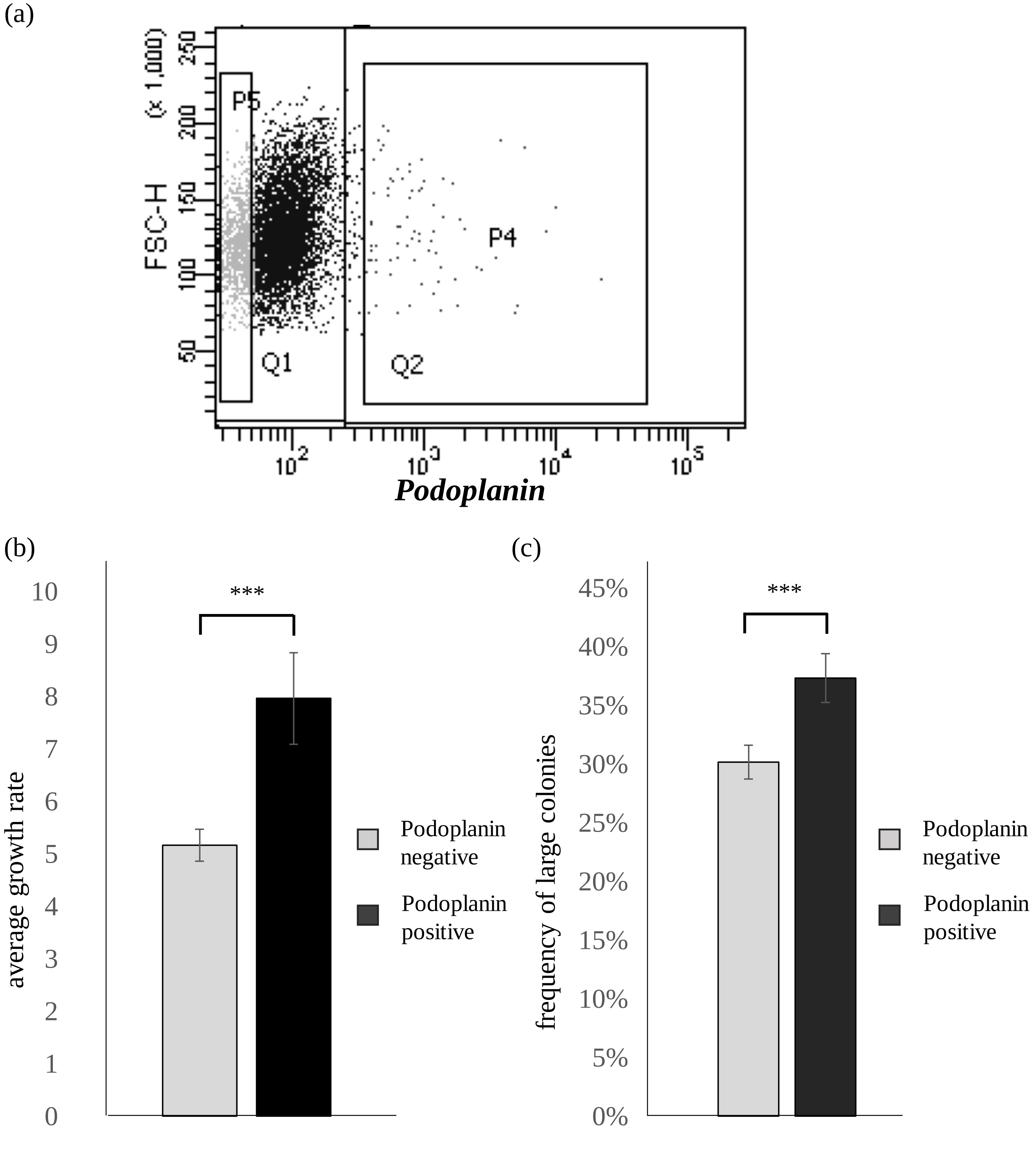

Supplementary Figure 2

Colony forming ability of single $PDPN^+$ and $PDPN^-$ NCIH2170 cell

(a) PDPN expression of NCIH2170 cell. PDPN positive fraction (P4) and PDPN negative fraction (P5) were sorted into 384 well plate. (b) The average growth rate of single $PDPN^+$ and $PDPN^-$ NCIH2170 cell. (c) The frequency of large colonies (≧8 cells) derived from single $PDPN^+$ and $PDPN^-$ NCIH2170 cells. ***$p < 0.01$

(a) G_1 phase

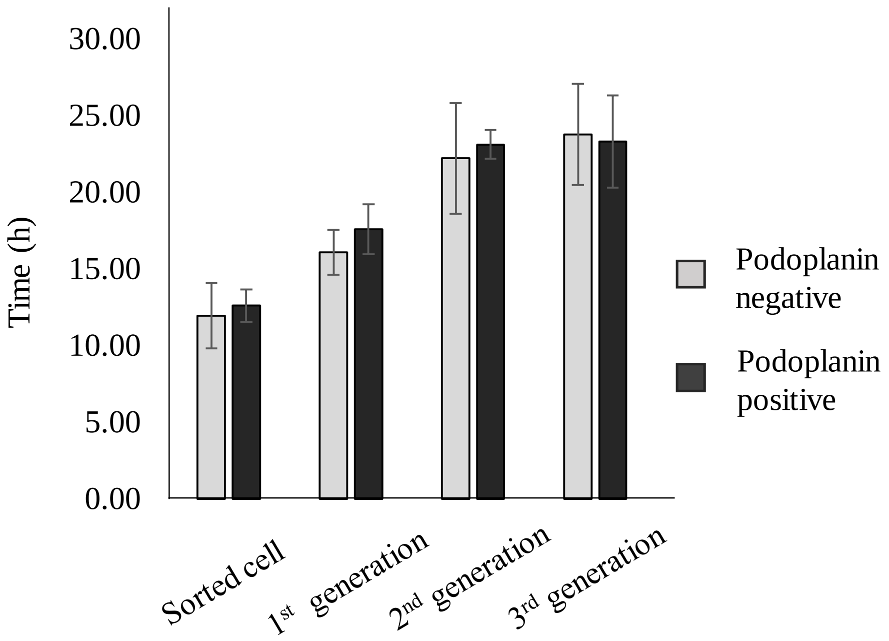

(b) S / G2 / M phase

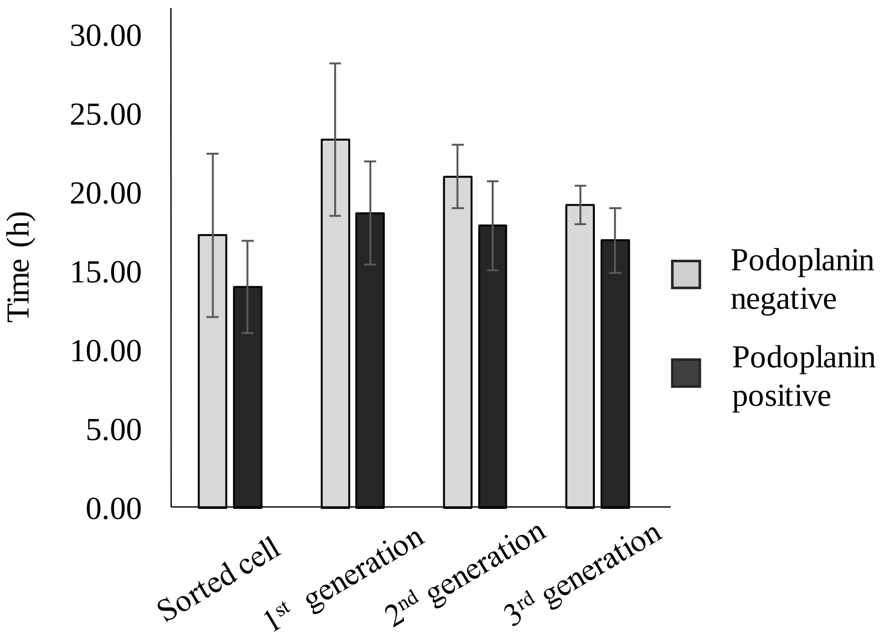

Supplementary Figure 3

Cell cycle status of $PDPN^+$- and $PDPN^-$-derived cells of each generation

(a) The G1 phase duration of each generation of cells derived from $PDPN^+$ (black bar) $PDPN^-$ (gray bar) single cells. (b) The length of the S/G2/M phases of each generation.

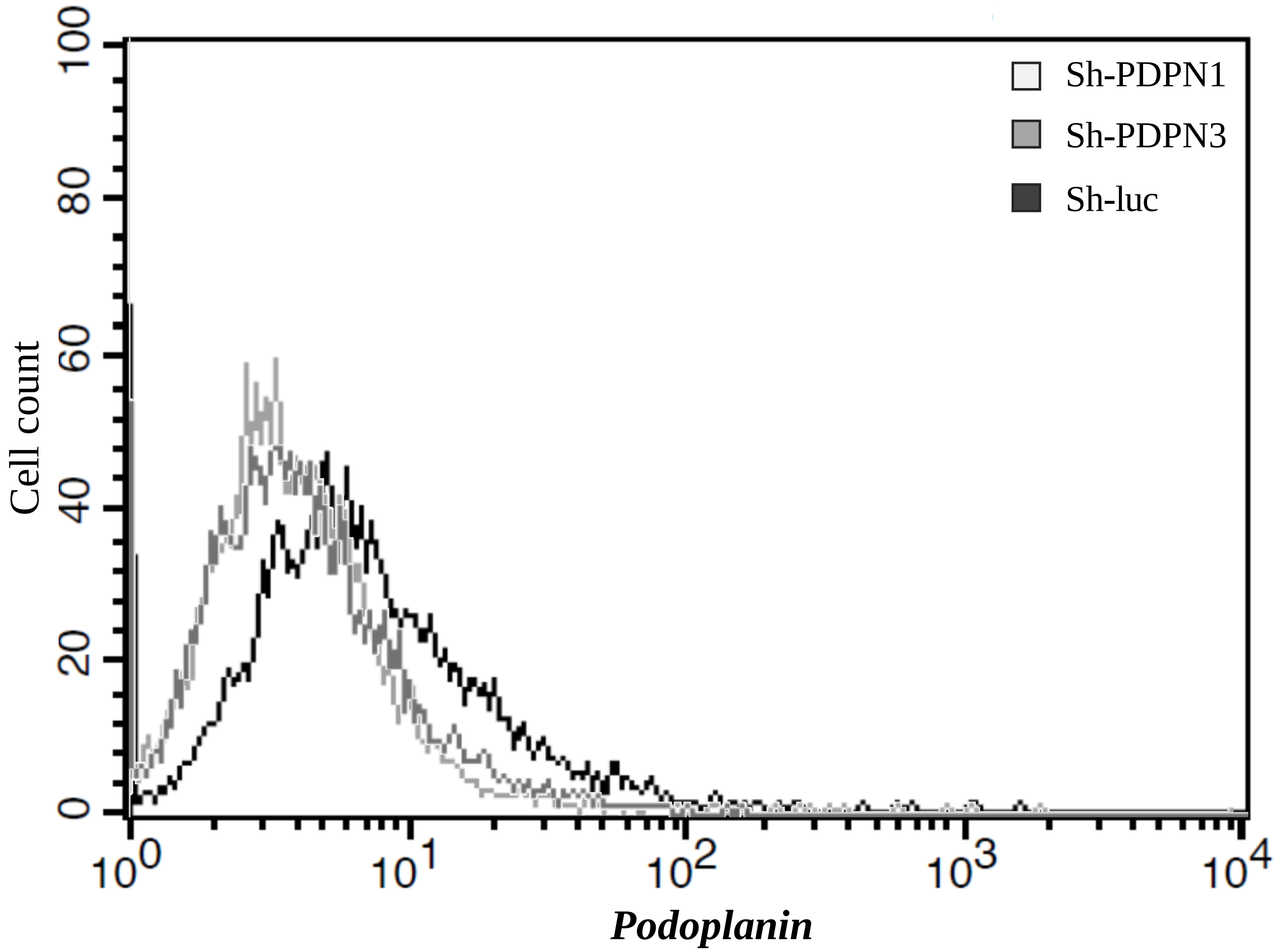

Supplementary Figure 4

PDPN expression of shRNA transfected A431 cells

The levels of shLuc- (controls), shPDPN1-, and shPDPN3- transfected cells are represented by the black, light gray, and dark grey lines, respectively.

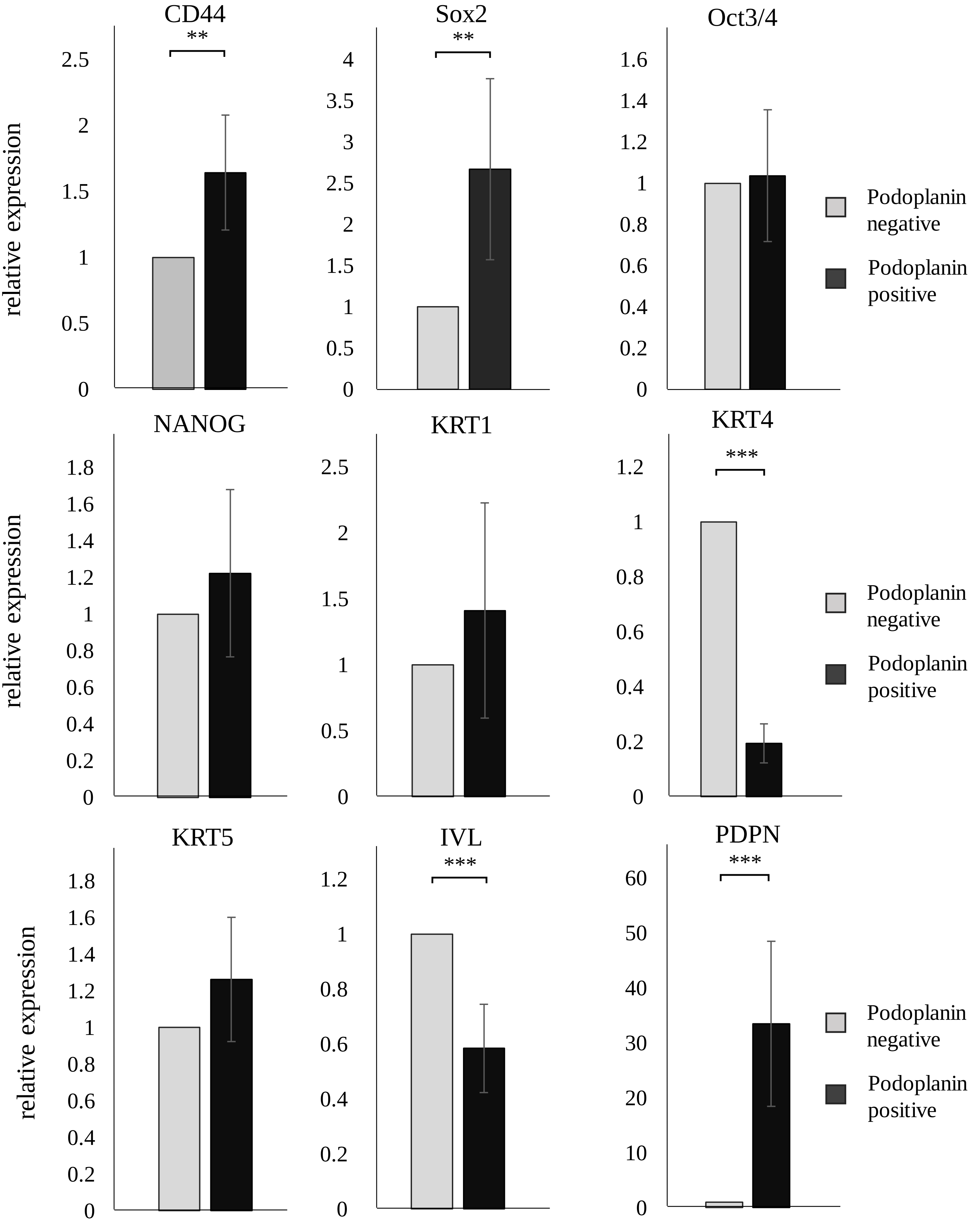

Supplementary Figure 5

Comparison of mRNA levels between PDPN$^+$ and PDPN$^-$ A431 cells

Y-axis represents relative expression ratios of PDPN$^+$ to PDPN$^-$ cells. $^{**}p < 0.05$, $^{***}p < 0.01$

Sup Table. 1 The number of seeded cells and grown cells in each experiment

**Experiment 1**	**Seeded cells**	**Number of grown cells**
Podoplanin^{-}	66	75
Podoplanin^{+}	40	155
Total	106	230

**Experiment 2**	**Seeded cells**	**Number of grown cells**
Podoplanin^{-}	45	18
Podoplanin^{+}	81	104
total	126	122

**Experiment 3**	**Seeded cells**	**Number of grown cells**
Podoplanin^{-}	42	122
Podoplanin^{+}	48	320
total	90	442

**Experiment 4**	**Seeded cells**	**Number of grown cells**
Podoplanin^{-}	46	93
Podoplanin^{+}	52	211
total	98	304

**Experiment 5**	**Seeded cells**	**Number of grown cells**
Podoplanin^{-}	54	55
Podoplanin^{+}	51	242
total	105	297

Sup Table. 2 The number of seeded cells and grown cells in each experiment of sh-RNA transfected cell

Experiment 1	Seeded cells	Number of grown cells
Sh-luc	29	82
Sh-PDPN1	30	21
Sh-PDPN3	45	50

Experiment 2	Seeded cells	Number of grown cells
Sh-luc	35	74
Sh-PDPN1	32	2
Sh-PDPN3	32	10

Experiment 3	Seeded cells	Number of grown cells
Sh-luc	53	113
Sh-PDPN1	48	22
Sh-PDPN3	43	74

Sup Table. 3 The number of seeded cells and grown cells in each experiment of ROCK inhibitor treated cells

Experiment 1	Seeded cells	Number of grown cells
PDPN (+) ROCK inhibitor (-)	24	118
PDPN (+) ROCK inhibitor (+)	25	91
PDPN (-) ROCK inhibitor (-)	30	65
PDPN (-) ROCK inhibitor (+)	18	40

Experiment 2	Seeded cells	Number of grown cells
PDPN (+) ROCK inhibitor (-)	58	235
PDPN (+) ROCK inhibitor (+)	61	199
PDPN (-) ROCK inhibitor (-)	40	151
PDPN (-) ROCK inhibitor (+)	54	89

Experiment 3	Seeded cells	Number of grown cells
PDPN (+) ROCK inhibitor (-)	68	283
PDPN (+) ROCK inhibitor (+)	57	196
PDPN (-) ROCK inhibitor (-)	41	95
PDPN (-) ROCK inhibitor (+)	65	73

Sup Table. 4 Primer information for qRT-PCR.

	Forward primer	Reverse primer
CD44	5’-CTCCGGACACCATGGACAA-3’	5’-CCACGTGGAATACACCTGCAA-3’
POU5F1	5’-GCTGGATGTCAGGGCTCTTTG-3’	5’-TTCAAGAGATTTATCGAGCACCTTC-3’
SOX2	5’-CTCCGGACACCATGGACAA-3’	5’-CCACGTGGAATACACCTGCAA-3’
NANOG	5’-CCTGTGATTTGTGGGCCTGA-3’	5’-CTCTGCAGAAGTGGGTTGTTTG-3’
IVL	5’-TTCTAAGATGTCCCAGCAACACAC-3’	5’-GTTTCATTGCTCCTGATGGGTA-3’
KRT1	5’-GGGAGCAAATCAAGTCACTCAAC-3’	5’-TCTGCTGCTCCAGGAACCTC-3’
KRT4	5’-CCATCAACCAGAGCTTGCTCAC-3’	5’-TCCATTTGGTCTCCAGGACCTTA-3’
KRT5	5’-CCCTCAACAATAAGTTTGCCTCCT-3’	5’-AGAACCTTGTTCTGCTGCTCCA-3’
PDPN	5’-GAAGACCGCTATAAGTCTGGCTTGA-3’	5’-ACTTTGTTCTTGCGCGTGGA-3’
GAPDH	5’-GCACCGTCAAGGCTGAGAAC-3’	5’-TGGTGAAGACGCCAGTGGA-3’
